# Insights into the spatial ecology of severely injured free‐living felids: Iberian lynx, bobcat, and snow leopard

**DOI:** 10.1002/ece3.11000

**Published:** 2024-02-16

**Authors:** Fernando Nájera, Stella F. Uiterwaal, Elena Crespo, Rebeca Grande‐Gómez, Juan Francisco Sánchez, Manuel Mata‐Huete, Jamie Palmer, Gabone Iturrarte, Jorge Peña, Bayaraa Munkhtsog, Bariushaa Munkhtsog, Andrey D. Poyarkov, Jose A. Hernandez‐Blanco, Dmitry Y. Alexandrov, Naranbaatar Galsandorj, Sharon L. Deem

**Affiliations:** ^1^ Karen C. Drayer Wildlife Health Center, School of Veterinary Medicine, University of California Davis California USA; ^2^ Saint Louis Zoo Institute for Conservation Medicine Saint Louis Missouri USA; ^3^ Asistencia Técnica de la Dirección General del Medio Natural y Desarrollo Sostenible de la Junta de Comunidades de Castilla‐La Mancha Toledo Spain; ^4^ Living Earth Collaborative Washington University in St. Louis Saint Louis Missouri USA; ^5^ Department of Biology Saint Louis University Saint Louis Missouri USA; ^6^ National Great Rivers Research and Education Center East Alton Illinois USA; ^7^ Fomento de Técnicas Extremeñas Badajoz Spain; ^8^ Gestión Pública de Extremadura Mérida Spain; ^9^ Institute of Biology, Mongolian Academy of Sciences Ulaanbaatar Mongolia; ^10^ Wildlife Institute, Beijing Forestry University Beijing China; ^11^ Irbis Mongolia Center Ulaanbaatar Mongolia; ^12^ A.N. Severtsov Institute of Ecology and Evolution Russian Academy of Sciences Moscow Russia

**Keywords:** amputation, felid, home range, *Lynx pardinus*, *Lynx rufus*, *Panthera uncia*

## Abstract

Severe musculoskeletal diseases, such as those associated with congenital or traumatic events, that result in missing limbs may compromise the fitness and survival of free‐living felids. Here we report the space use of four amputee individuals from three felid species captured from 2017 to 2022 in Missouri (USA), Toledo and Badajoz (Spain), and Suitai Khairkhan Mountain (Mongolia). We describe home ranges and daily travel distances post‐release of free‐living felids that had either suffered a traumatic amputation or following a surgical amputation. We compared these data with those reported in the literature for felids without amputations. Forelimb or hindlimb amputation did not affect the hunting, mating, or territory patrolling behavior of any of the individuals. However, we recorded significant differences in the daily movement before and after the traumatic event of the Iberian lynx forelimb amputee. We attribute this difference to the physical impairment, although we consider other variables that may have played a role. Nevertheless, all animals appeared to cope well with their limb loss, showing home ranges and daily distances within those recorded for their sex and species. Unless amputee felids represent a threat to domestic livestock or humans, our data suggest these individuals may remain free‐living as they contribute to local population persistence and appear to maintain good general health and welfare.

## Graphical abstract

As a result of anthropogenic pressures, wild felids worldwide may suffer from severe lesions that may impact their overall fitness, behavior, and/or survival. Despite the severity of skeletal trauma that resulted in limb amputation, two Iberian lynxes, one bobcat, and one snow leopard tagged with telemetry collars didn't show differences in movement patterns compared with healthy conspecifics, highlighting the resilience, adaptation, and plasticity of these individuals.

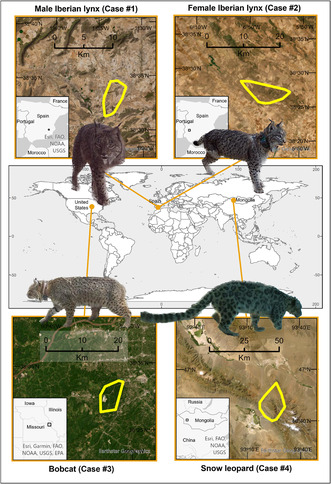

## 1 INTRODUCTION

Carnivores are important members of many ecosystems, structuring communities and landscapes through direct and indirect trophic interactions (DeLong, 2021; Ripple et al., 2014). Many of these ecologically important species have experienced recent decline due to habitat destruction, prey depletion, and poaching (Loveridge et al., 2020; Ripple et al., 2014; Wolf & Ripple, 2016). By understanding the conditions under which individuals of these species can successfully free‐range, exhibit natural behaviors, and contribute to population growth, we can help conserve these carnivores and mitigate the ecological impacts of their decline.

Many carnivores face traumatic injury or death from threats such as vehicle collisions or trapping. In felids, such injuries include an entire chest laceration due to a wire snare in an African leopard (*Panthera pardus*) (Power et al., 2020), skin wounds and a canine fracture due to a vehicle collision in a puma (*Puma concolor*) (Adania et al., 2017), long‐bone fractures due to vehicular trauma‐ or capture‐related injuries in Florida panthers (*Puma concolor coryi*) (Yong et al., 2018), a closed, complete, non‐comminuted transverse fracture of the left radius and ulna due to a leg snare in a Canada lynx (*Lynx canadensis*) (Poole et al., 1998), a femur fracture in a jungle cat (*Felis chaus*) (Ali, Basumatary, & Boruah, 2015) and an Iberian lynx (*Lynx pardinus*) (Rodriguez et al., 1995), and an injury resulting in evisceration of a small intestinal loop in a clouded leopard (*Neofelis nebulosa*) (Ali, Basumatary, & Choudhury, 2015). Carnivores that have suffered from such traumatic injuries may be rehabilitated and released to continue contributing to wild populations, but the success of these releases is unclear.

Limb amputation (a salvage surgical procedure to treat severe trauma, extensive nerve injury, malignant neoplasia, severe infection, ischemia, necrosis of the limb, severe disability, and congenital disorders) (Ferreira et al., 2019; Gabriel et al., 2017; Kim et al., 2020) may particularly impact individuals' long‐term fitness and survival (Argyros & Roth, 2016). Although rehabilitation and release or observation of carnivores that experienced a limb amputation has been described (e.g., river otter (*Lontra canadensis*), Kellnhauser, 1970; Iberian wolf (*Canis lupus signatus*), Rio‐Maior et al., 2016; raccoon dog (*Nyctereutes procyonoides*), Kim et al., 2020; Asiatic black bear (*Ursus thibetanus*), Jeong et al., 2021), there are no available reports of the rehabilitation, release, post‐release survival or movement patterns of amputee free‐living felids. Data extracted from museum specimens revealed that animals may be able to survive and reproduce even after a traumatic limb amputation (Garcia‐Perea, 2000). However, data obtained from museum collections, although extremely valuable, are unable to provide data on behavior of amputee individuals.

To evaluate the success of released carnivores recovered from severe injuries, investigation of at least the post‐release survival and movement patterns is essential (Rio‐Maior et al., 2016). Movement is fundamental to carnivores' ability to find and capture food, avoid competitors or predators, find mates, scent mark, and otherwise communicate with conspecifics (Powell, 2012). One of the best methods to document movement is by fitting a tracking device to the individual (e.g., GPS telemetry collar, Rio‐Maior et al., 2016; VHF radio collar, Adania et al., 2017; ear tag radio transmitter, Jeong et al., 2021).

We report the spatial data collected from four free‐living felids: two Iberian lynx (*Lynx pardinus*, IUCN status: Endangered), a bobcat (*Lynx rufus*, IUCN status: Least concern), and a snow leopard (*Panthera uncia*, IUCN status: Vulnerable), along with the rehabilitation process in the Iberian lynxes. In an attempt to assess overall fitness in these injured felids, our study aimed to address two main research questions: (i) What was the home‐range size of our study subjects and how do they compare to conspecifics found in the literature? and (ii) What was the average distance traveled within a 24‐h monitoring period by our study subjects and how do they compare to conspecifics reported in the literature? We also included the home range and daily distances traveled when the individuals were physically healthy prior to their traumatic injuries (for the Iberian lynx and snow leopard) or for a four‐legged conspecific in the same area (for the bobcat). We hypothesize that the physical impairment of amputee individuals is reflected in their movement patterns: a smaller species‐specific size home range and less daily distance traveled compared to overall healthy conspecifics or pre‐amputation in those individuals with paired data.

We further discuss the success of these individuals in foraging for prey and interacting with mates, two important components of fitness.

## 2 METHODS

### 2.1 Data collection

We present data on four amputee free‐living felids: a male Iberian lynx (who was rescued, rehabilitated, and released after vehicular trauma who suffered from neurological lesions that needed surgical amputation in a forelimb); a snow leopard (who was captured for research purposes suffering from a recent traumatic amputation caused by a leg hold trap/snare); a bobcat (who was also captured for research purposes with a recently self‐healed hindlimb traumatic amputation); and a female Iberian lynx rescued with hindlimb injury that underwent a surgical amputation due to the severity of the lesion.

**FIGURE 1:**
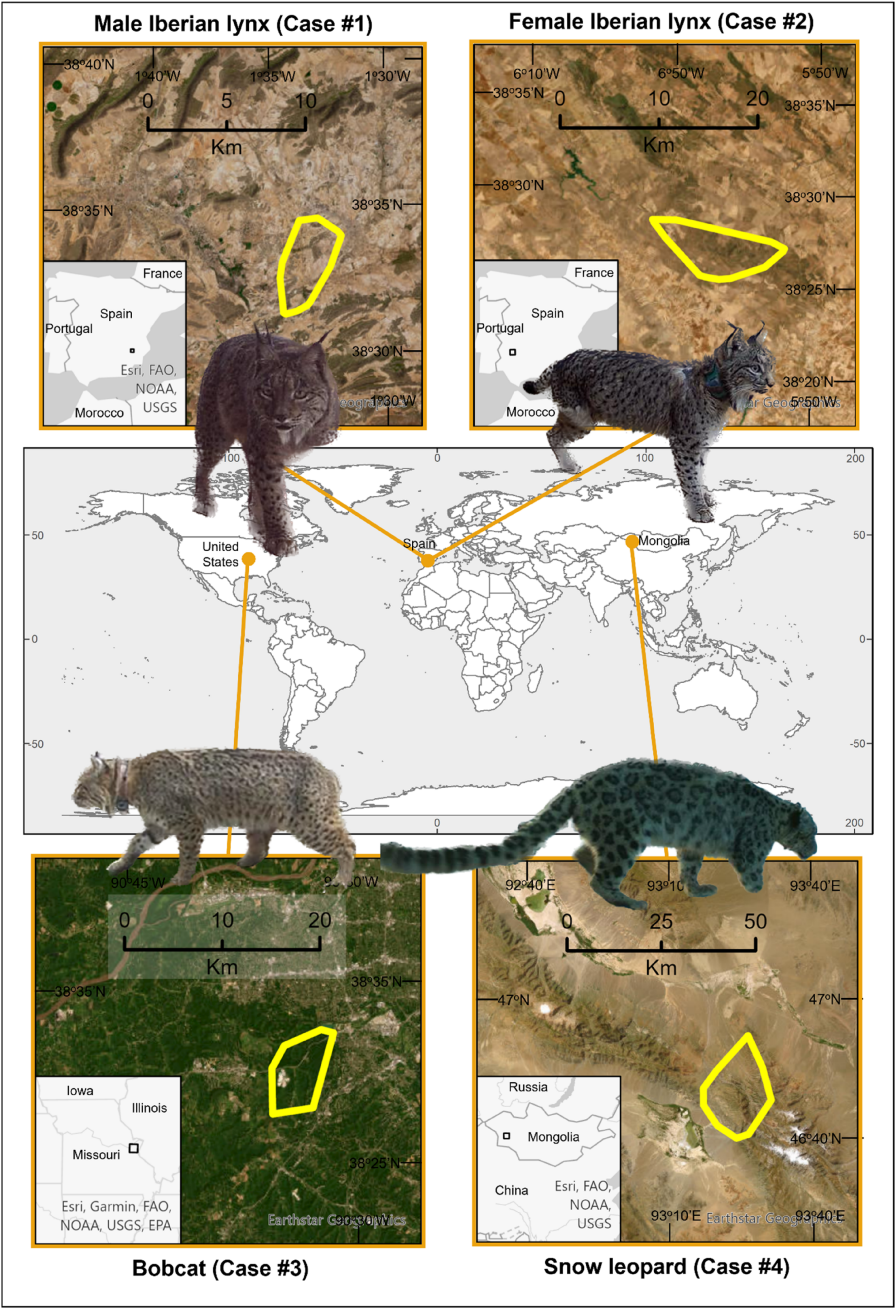
Locations of the four sites of this study on the movement and health of three‐legged free‐living felids. Case #1: Toledo and Albacete (Spain), Case #2: Badajoz (Spain), Case #3: Missouri (USA), and Case #4: Sutai (Mongolia).

Data originate from three research studies carried out on these felid species (Figure 1). The Iberian lynx cases correspond to European Union‐funded projects aiming to restore Iberian lynx populations over their entire historical distribution and to create a genetically and demographically functional Iberian lynx metapopulation. The bobcat case is part of a project in the USA aiming to investigate the spatial epidemiology and ecology of Missouri native carnivores in two distinct landscapes. Lastly, the snow leopard case is from a project aiming to determine abundance and density of snow leopards, assess habitat suitability, and estimate the home ranges and diet of snow leopards in the Sutai Khairkhan mountains (Govi‐Altai provinces) of Mongolia. Study site descriptions are given in Appendix S1, and detailed case descriptions are given in Appendix S2.

#### 2.1.1 Case #1: Male Iberian lynx

In July 2015, an adult male Iberian lynx was released as a part of the Iberian Lynx Reintroduction Program in Toledo, Spain. The lynx was fitted with a GPS‐GSM collar (Microsensory, Fernán Núñez, Spain; locations every 4 h) which functioned until January 7, 2018. Around July 22, 2019, the lynx was hit by a vehicle and the collar was destroyed. Camera traps (Moultrie W‐900, Birmingham, AL) in the area showed the lynx 1 week later, limping on the left forelimb. On August 15, 2019, the lynx was accidentally trapped in a chicken coop and immobilized. An initial health assessment in the field revealed a severe skin abrasion in the dorsal aspect of the left forelimb, and complicated crown fractures of the mandibular and maxillary left canines. The medical history, clinical presentation, and neurological examination led to a tentative diagnosis of caudal brachial plexus avulsion of the left forelimb. Poor prognosis for the affected limb resulted in a decision to perform a surgical amputation of the proximal half of the humerus and the lynx was further rehabilitated. On October 26, 2021, we fitted the lynx with a VHF‐GPS‐GSM collar (Microsensory, Fernán Núñez, Spain; locations every 4 h) and released the individual. To decrease explorative behavior post‐release, we provided domestic rabbits for 8 weeks via a supplementary feeding station, regularly decreasing the number of rabbits provided over the last 4 weeks to avoid dependence on the feeding station. On February 6, 2022, the mortality signal was triggered on the lynx's collar, and the individual was found drowned in a human‐made irrigation pond. A necropsy was performed. Table 1 summarizes the main events that occurred during the rehabilitation process of this Iberian lynx.

**TABLE 1:** Events and dates during the rehabilitation and release of a male Iberian lynx in Castilla‐La Mancha, Spain.

Event	Date	Total days
Vehicle collision	22 July 2019	0
First capture	15 August 2019	23
Entry wildlife rehabilitation center—quarantine	15 August 2019	23
Physical and radiographic examinations	5 September 2019	44
Surgery	18 October 2019	87
Introduction of live prey	25 October 2019	94
Move to secondary enclosure	1 May 2021	650
Physical examination, biological sampling, and radio‐collaring prior to release	26 October 2021	826
Release	16 November 2021	846
Encounter with female	22 November 2021	852
Mating	12–20 December 2021 (estimated)	873–881
Death	6 February 2022	926

#### 2.1.2 Case #2: Female Iberian lynx

The second Iberian lynx was a yearling wild‐born female Iberian lynx. On February 22, 2019, we video‐recorded the individual limping within the boundaries of the reintroduction area of the Hornachos‐Matachel Valley. The individual's gait and right hindlimb appearance resembled a severe fracture. A review of the camera‐trap footage revealed that the individual first showed this lesion at least 20 days prior. The field team successfully captured the lynx via cage traps within 2 days and emergency surgery was performed the same day. Radiological examination exposed an open fracture of the right tibia and fibula, with necrosis of the right tarsus. Physical examination also revealed skin abrasions in several regions and several frayed claws, all compatible with a vehicle collision. Due to the grave prognosis for functional use in the affected limb, we performed surgical amputation distal to the femur. On April 5, 2019, we fitted the lynx with a VHF radio collar (Q‐4, Andreas Wagener, Köln, Germany) before releasing her back to the capture site. At this time, she weighed 6 kg. Once released, we monitored the animal via camera traps and using telemetry performed 1–3 days per week. Locations were obtained by triangulation using a 3‐element Yagi antenna (RA‐23, Telonics, Mesa, AZ) and hand‐held radio telemetry receiver (ICOM America, Kirkland, WA) (see Rueda et al., 2021).

On June 28, 2022, we captured the lynx for a routine health assessment and changed the radio collar (Q‐7, Andreas Wagener, Köln, Germany). Table 2 summarizes the main events that occurred during the rehabilitation process of this female Iberian lynx.

**TABLE 2:** Events and dates during the rehabilitation and release of the female Iberian lynx in Extremadura (Spain).

Event	Date	Total days (estimated)
Acquired lesion	February 2, 2019 (estimated)	0
First sighting of limping	February 22, 2019	20
First capture	February 24, 2019	22
Physical examination and surgical amputation	February 24, 2019	22
Entry quarantine with live prey provided	February 24, 2019	22
Physical examination and radio‐collaring prior to release	April 5, 2019	62
Release	April 5, 2019	62
Second capture	June 28, 2022	146
First encounter with male	November 2022 (estimated)	272

#### 2.1.3 Case #3: Bobcat

On February 9, 2022, we captured an adult male bobcat in Missouri, USA in a cage trap baited with road‐killed white‐tailed deer. We anesthetized the bobcat and performed a routine physical examination revealing a missing right limb below the tibiotarsal joint. We suspect the lesion may have been from a leg hold trap capture since bobcats are considered furbearers in the state of Missouri and trapping is legally permitted (Missouri Department of Conservation, 2021). The bobcat weighed 10.5 kg. We fitted the bobcat with a GPS logger (W500, Advance Telemetry Systems, Isanti, Minnesota, USA; locations every 4 h) and we released him at the site of capture the same day. After 1 month of tracking, we lost the signal of the individual. One week later, the bobcat was photographed by a camera trap in a private estate 20 km southeast of the capture site. Despite our efforts of terrestrial and aerial telemetry, we were unable to locate the individual again. To allow a comparison between two adult males from the same area but with different conformation, we include a second adult male with all four limbs intact, captured 1 week earlier at the same site, in this study. Capture, anesthesia, and tagging protocols were the same for both bobcats.

#### 2.1.4 Case #4: Snow leopard

On November 12, 2016, we captured and collared (Lotek GPS‐Argos collar, Lotek Wireless Inc., Ontario, Canada) an adult snow leopard with no serious injuries. The collar provided locations only until March 20, 2017. On October 23, 2017, we recaptured the leopard with an Aldrich snare. The leopard weighed 44 kg. A physical examination revealed that the right forelimb was missing below the proximal humerus. We sutured the skin flap to protect the muscle and bone structures. This individual also presented a separate, fresh wound of 5.6 mm diameter located in the left lateral caudal abdomen, compatible with gunshot. We fitted him with a GPS‐Iridium collar (Lotek Iridium LiteTrack 420, Lotek Wireless Inc., Ontario, Canada; locations every 2 h) which functioned until July 5, 2018.

### 2.2 Data analyses

We calculated the home range of each study subject by calculating the minimum convex polygon (MCP) and kernel density estimates (KDEs) using the *adehabitatHR* package (Calenge, 2006). We chose MCP since it is a commonly used, simple measure that allows comparison with previous home‐range estimates found in the literature. We calculated the 100% MCP (which represents the smallest convex polygon for all the study individuals' locations), 95% MCP (the smallest convex polygon after removing 5% of the outliers from all the locations), and 50% MCP (the smallest convex polygon after removing 50% of the outliers of the dataset). We also calculated KDEs, since this home range is often presented in the literature for our study species and since KDEs tend to be better for intraspecific comparisons of home ranges than MCPs (Nilsen et al., 2008). KDEs calculate the harmonic mean and create isopleths of the intensity of home‐range utilization (Powell, 2000). We estimated KDE using the 95% and 50% probability contours, using *href* as bandwidth. We defined the core area as the 50% isopleth of the kernel density estimator. Since recent snow leopard research refers to the local convex hull (LoCoH) as a more biologically appropriate home‐range estimate for this species (see Johansson et al., 2016; Rosenbaum et al., 2023), we also included this index in our snow leopard case. For this case, and after testing a range of values between 20 and 25, we selected *a* = 24.1 km, as the distance parameter, since *a* = 21 km (used by Rosenbaum et al., 2023) did not fit our snow leopard data.

Lastly, for all individuals tracked with GPS collars (male Iberian lynx, bobcats, snow leopard) we used the *adehabitatLT* package to calculate daily distance traveled (from noon to noon) for all days for which we had a complete set of daily fixes (i.e., 7 points, one point every 4 h for 24 h) and we report the means and ranges. For the individuals for which we had both pre‐ and post‐amputation location data (the male Iberian lynx and the snow leopard), we performed *t*‐tests to determine whether daily distance traveled differed after amputation. For the snow leopard, we also compared daily distance traveled in a 14‐day period immediately after release and in a 28‐day period 3 months post‐release, to determine whether distance traveled is subject to a habituation period post‐release and whether this differed before and after amputation. We did not perform this analysis for the male Iberian lynx (due to sparse location data shortly after release both pre‐and post‐amputation) or the bobcat (because we had location data for just ~1 month). For all analyses, we used locations obtained every 4 h; if fixes were taken more frequently than once every 4 h, we removed “extra” fixes. We performed all analyses in R 4.2.2.

## 3 RESULTS

### 3.1 GPS data

After removing outlying, erroneous points from our GPS collars, we collected a total of 1992 fixes (pre‐amputation) and 403 fixes (post‐amputation) for the male Iberian lynx, 692 fixes (pre‐amputation) and 1184 fixes (post‐amputation) for the snow leopard, and 105 fixes for the bobcat. For comparison with the bobcat, we also recorded 1680 locations of a separate, four‐legged adult male bobcat whose range overlapped with the amputee bobcat. The average lifespan of the GPS collars was 214.25 days (SE: 54.6, range: 30–513 days). For the female Iberian lynx equipped with VHF collars, we collected 263 fixes over the course of 47 months.

### 3.2 Amputation and movement

**FIGURE 2:**
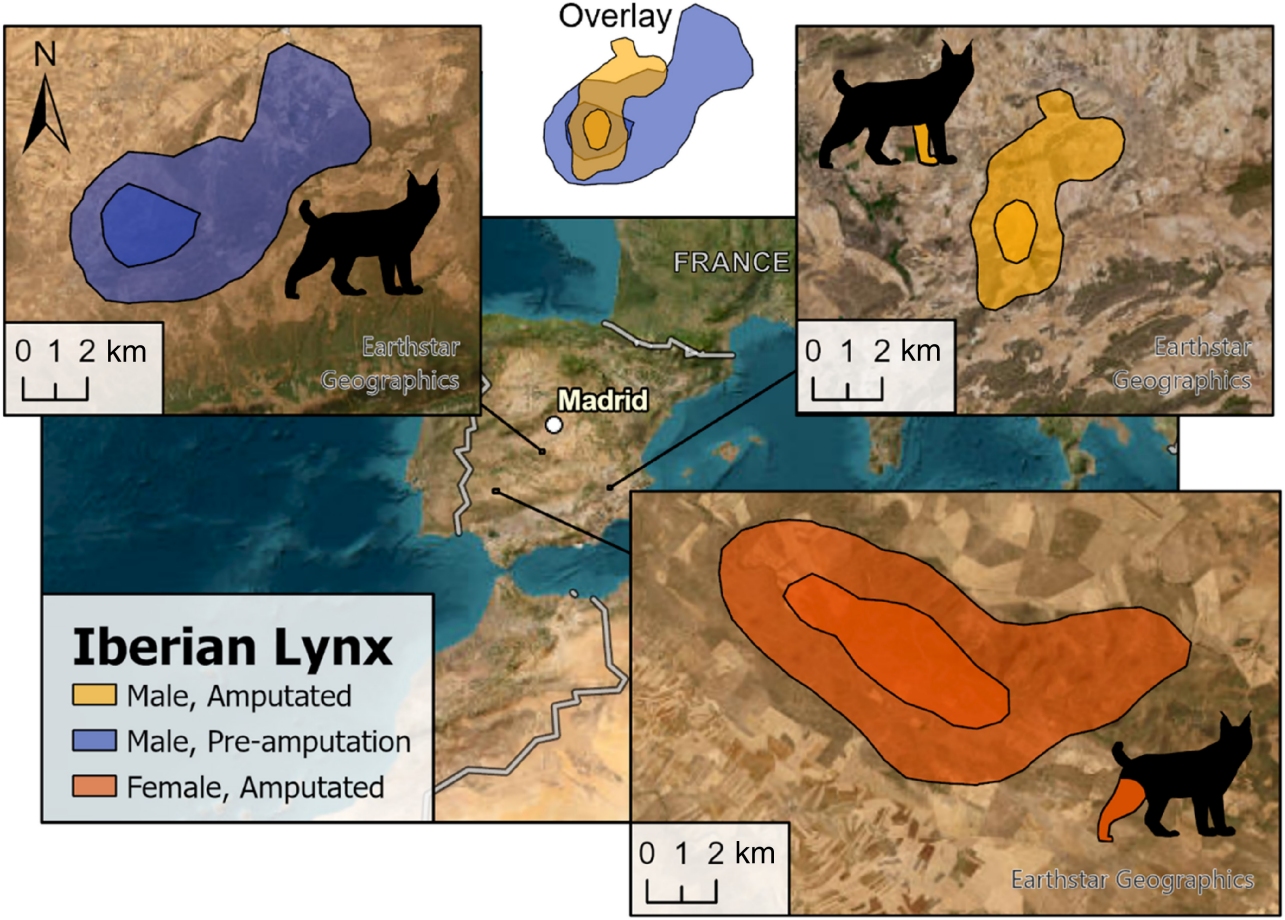
Pre‐ and post‐amputation 95% and 50% KDEs of the male Iberian lynx (Case #1) and post‐amputation 95% and 50% KDEs of the female Iberian lynx (Case #2) in Spain and in our study. For size comparison, an overlay of the male lynx's pre‐ and post‐amputation KDEs is shown. Silhouettes indicate the affected limb.

**FIGURE 3:**
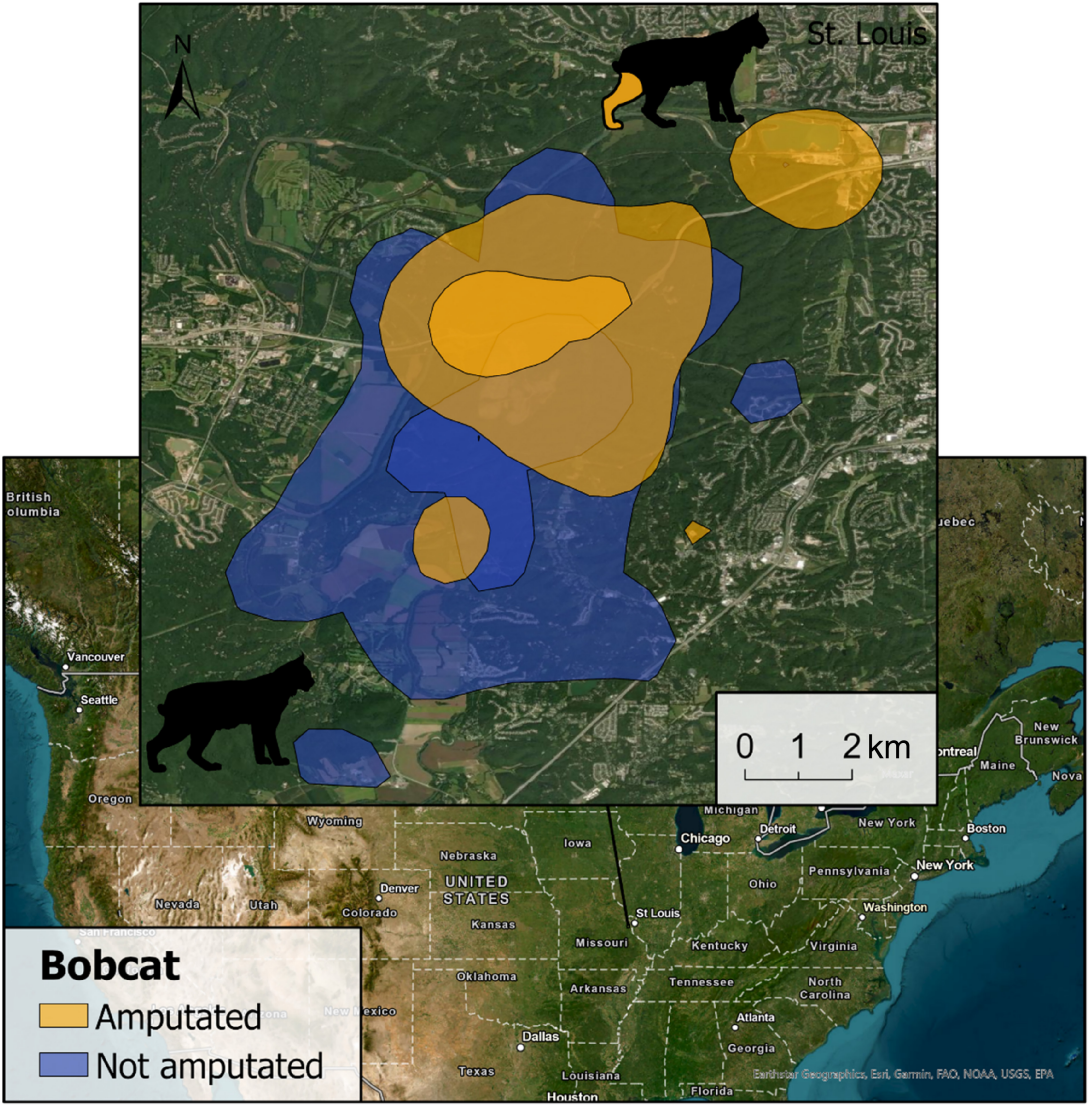
95% and 50% KDEs of the amputated (Case #3) and not amputated bobcats in Missouri, United States in our study. Silhouettes indicate the affected limb.

**FIGURE 4:**
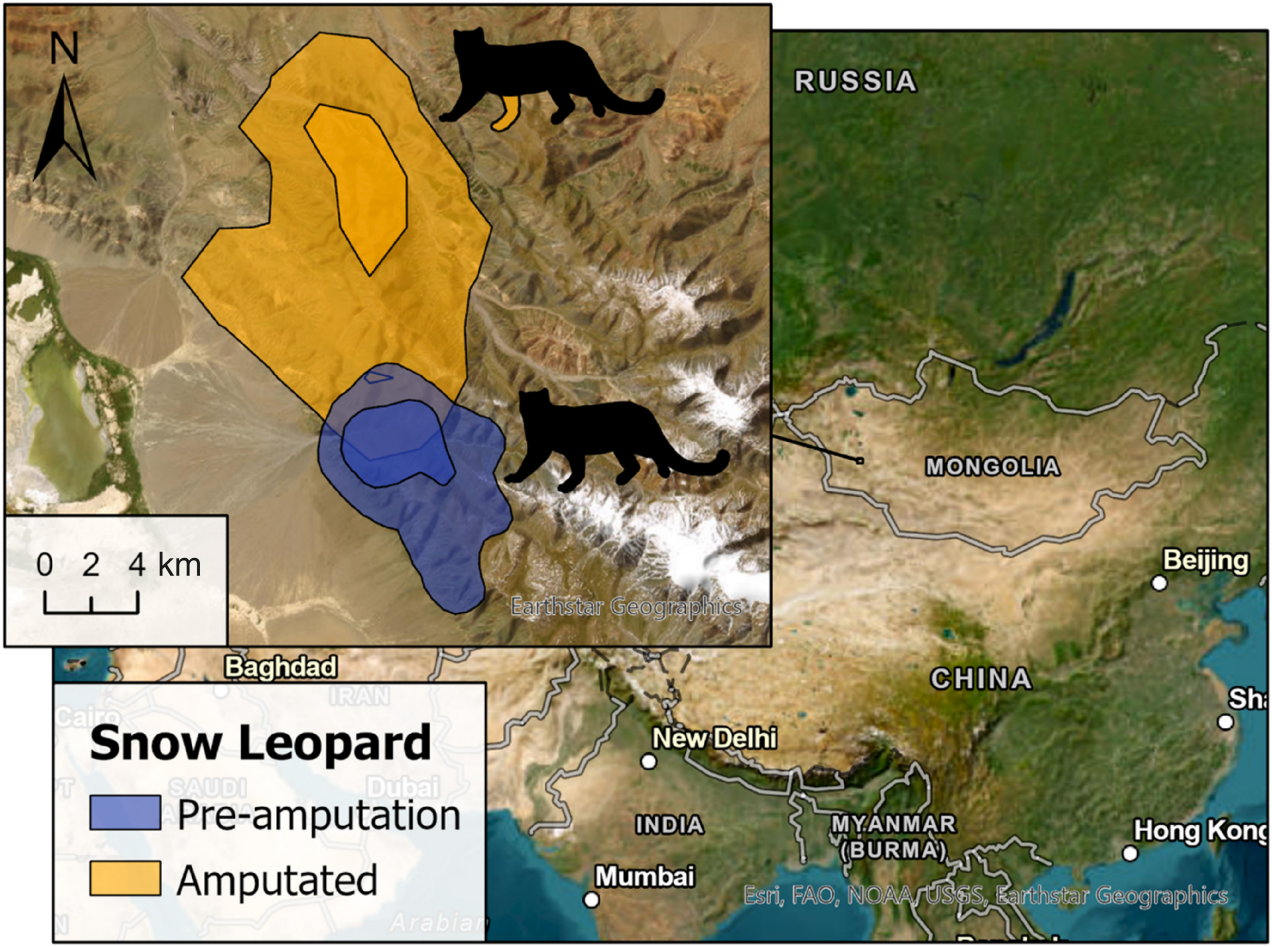
95% and 50% KDEs of the snow leopard (Case #4) in Mongolia in our study. Silhouettes indicate the affected limb.

**FIGURE 5:**
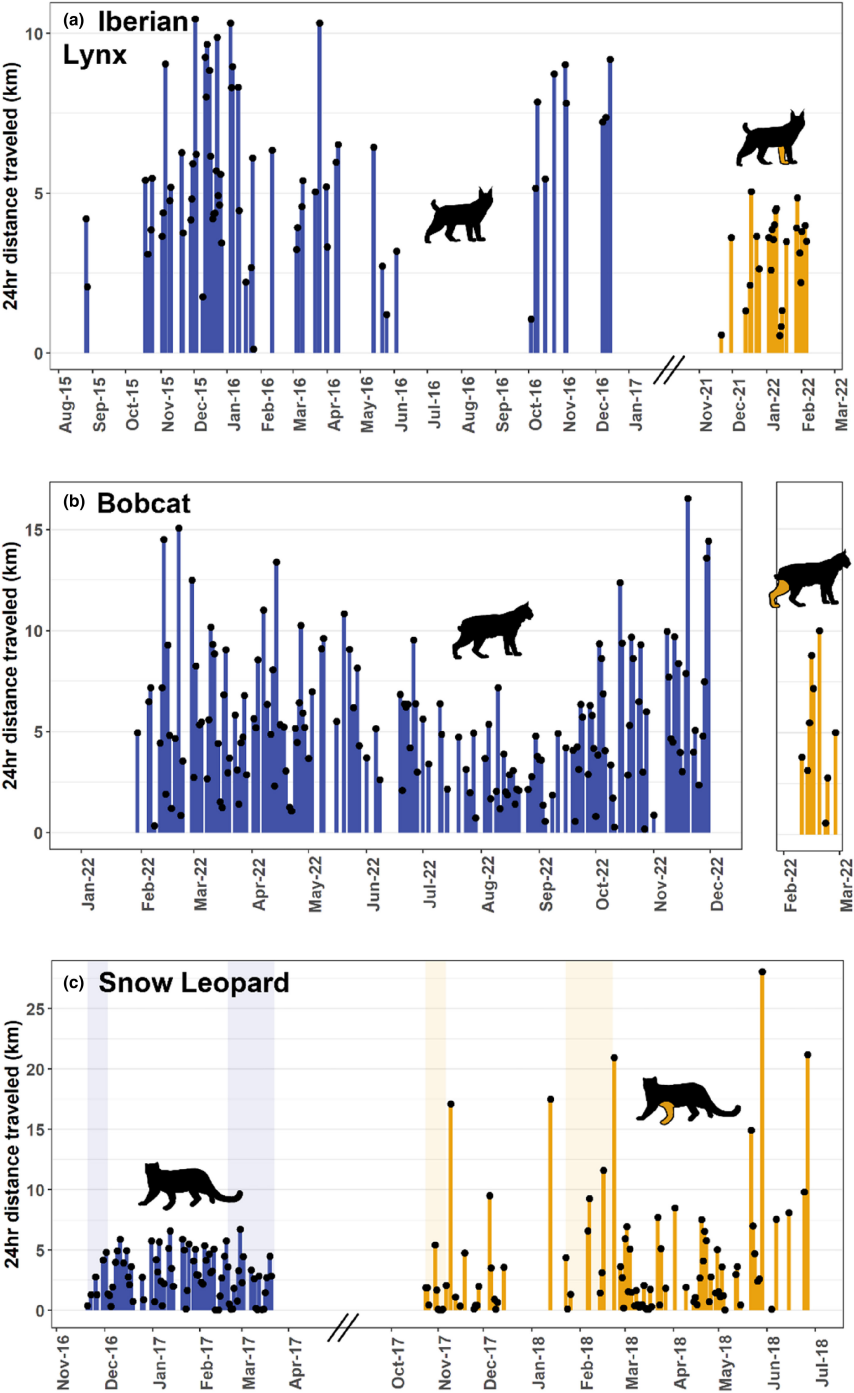
Daily distances traveled by (a) the male Iberian lynx in Case #1 before (purple) and after (orange) amputation, (b) the focal bobcat in Case #3 after amputation (orange) and a four‐legged sympatric male (purple), and (c) the snow leopard in Case #4 before (purple) and after (orange) amputation. Shaded areas in (c) highlight the first 14 days and the third month post‐release.

Home‐range estimates (MCP 100%, 95%, 50%; KDE 95%, 50%; and LoCoH 95%) and daily distances covered by amputee individuals were within the range of those recorded for their sex and species (Table 3 and Figures 2, 3, 4, 5). The daily distance traveled by the male Iberian lynx was significantly less after amputation compared to before amputation (*t*(67.7) = 6.22, *p* ≤ .001, Figure 5a). There was no difference in daily distance moved between the amputated and sympatric non‐amputated bobcats (*t*(9.2) = 0.09, *p* = .931, Figure 5b). In contrast, after amputation, the snow leopard's daily distance traveled showed an increase that approached significance (*t*(114.8) = −1.87, *p* = .063, Figure 5c).

**TABLE 3:** Home‐range estimates and daily distances in four cases of three‐legged free‐living felids in this study, and data obtained from the literature.

Species (case)	Daily distance (km) [mean, range]	Home range					LoCoH 95% (km^2^)	References
		MCP 100% (km^2^) [mean, range]	MCP 95% (km^2^) [mean, range]	MCP 50% (km^2^) [mean, range]	KDE 95% (km^2^) [mean, range]	KDE 50% (km^2^) [mean, range]		
Iberian lynx (male, pre‐amputation)	5.631 [0.122–10.446]	65.512	35.671	8.312	38.175	5.452	NA	
Iberian lynx (male, post‐amputation)	3.085 [0.547–5.048]	14.952	11.986	1.042	14.613	1.650	NA	
Iberian lynx (males)^a^	[6.6, 1.5–23.48]	NA	[9.08]	NA	[8.18, 1.84–13.37	[2.52, 0.4–5.76]	NA	Rueda et al. (2021) , Sarmento et al. (2019)
Iberian lynx (female, amputated)	NA	44.8	32.116	10.911	57.599	12.767	NA	
Iberian lynx (females)^a^	6.6, 1.5–23.48	NA	5.72	NA	6.34, 0.78–22.03	1.03, 0.21–2.19	NA	Rueda et al. (2021) , Sarmento et al. (2019)
Snow leopard (pre‐amputation)	2.879 [0.028–6.708]	49.690	40.235	12.533	53.935	13.762	32.106	
Snow leopard (post‐amputation)	3.947 [0.034–28.056]	298.350	127.878	35.720	135.737	22.934	60.022	
Snow leopard males^a^	0.5–10.8 [5.1, 0.5–10.8]	[101.5, 61–142]	[69–615]	NA	[88.6–617]	NA	[51.1–123.4]	Johansson et al. (2016), McCarthy et al. (2005), Rosenbaum et al. (2023)
Bobcat (amputated)	5.198 [0.564–10.011]	36.697	23.171	2.894	31.018	4.754	NA	
Bobcat male (overlapping)	5.284 [0.176–16.536]	96.527	57.864	10.522	62.515	11.890	NA	
Bobcat (males)^a^	[5.92, 4.1–7.1]^b^; [8.81, 5.8–11.7]^c^ [2.08, 0–8.9]^d^	[60.4, 29.8–107.5]^d^	[39.7, 2.86–167.9]^e^ [74.1]^d^	NA	NA	NA	NA	Ferguson et al. (2009), Kitchings and Story (1978), Newbury (2013), Hamilton (1982)

Abbreviation: NA, not available.

^a^Data obtained from the literature (see references: ^b^Ferguson et al. (2009); ^c^Kitchings and Story (1978); ^d^Hamilton (1982); ^e^Newbury (2013).

### 3.3 Movement after release

Prior to amputation, the snow leopard's mean daily distance traveled in the first 14 days after release was 1.44 km (95% CIs: −0.13 to 3.01). This was lower than the individual's mean daily distances after 3 months 2.11 km (95% CIs: 1.27–2.95), although overlapping confidence intervals suggest this increase is not significant. After amputation, the individual's mean daily distance traveled was 1.36 km (95% CIs: 0.17–2.56) in the first 14 days after release and 4.73 km (95% CIs: 1.30–8.16) in the third month after release, again showing no significant change in travel distance (Figure 5c).

### 3.4 Foraging

**FIGURE 6:**
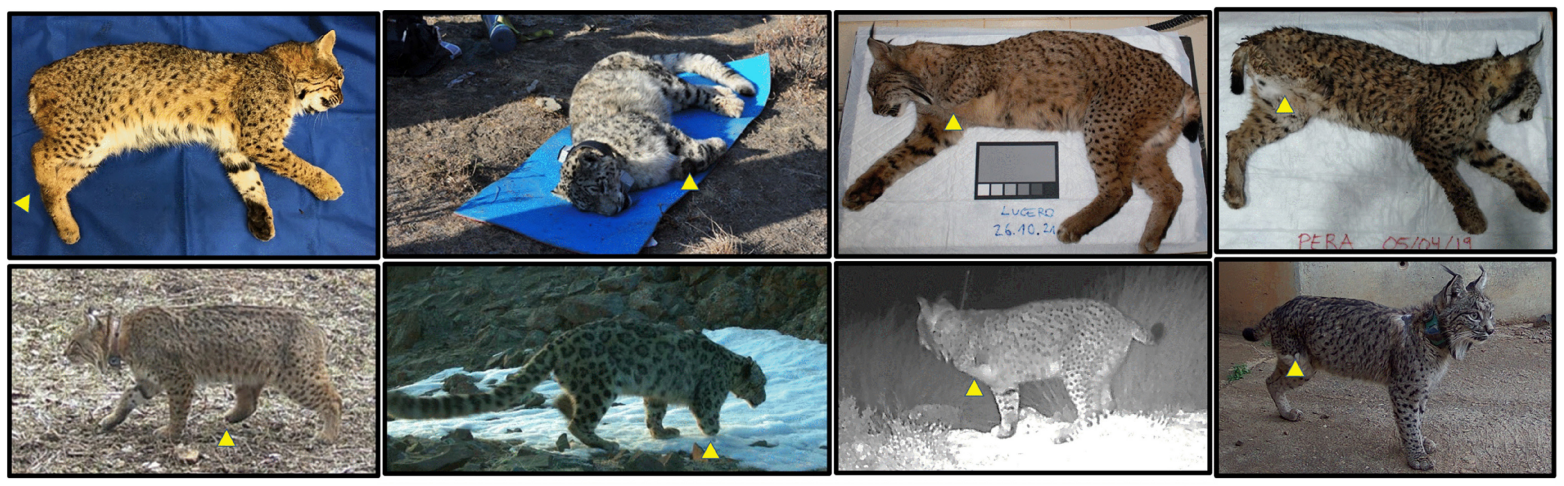
Amputee felids from this study. Top: anesthetized individuals shown in the study before release after amputation. The yellow triangle shows the missing limb. From left to right: bobcat from United States; snow leopard from Mongolia; Iberian lynx (male) from Spain; and Iberian lynx (female) from Spain.

During post‐surgery quarantine, the male Iberian lynx showed hunting skills (e.g., stalking, ambush, pursuit, and capture) in accordance with the species (Rivas et al., 2016). After release, we did not register any challenges in the individual's hunting skills once the feeding station was no longer in use. During necropsy, the lynx presented with an optimum body condition score and a weight of 14.4 kg, 2.5 kg higher than at release. The female Iberian lynx body condition improved over the ~20 days during which she was recorded on camera traps with the hindlimb lesion, meaning that the individual had already adapted to having this disability. During post‐surgery quarantine, the lynx's hunting skills on live prey were normal, and she did not show any signs of struggle while hunting. At the time of her second capture, the lynx presented with a good body condition score and body weight was 8.7 kg, 3.9 kg higher than at initial capture. The bobcat had an appropriate weight at capture (10.5 kg), and photos from this camera trap site revealed that the individual remained in good body condition (Figure 6). The snow leopard had a good body condition score and a weight (44 kg) within the normal range, despite the missing limb. This weight was similar to that recorded when the snow leopard was first captured in 2016 (46 kg). In addition, we photo‐captured the snow leopard in good body condition after the collar dropped off in April 2019 (Figure 6).

### 3.5 Mating

One week after the release of the male Iberian lynx (Case #1), we recorded a male–female interaction via camera trap photos. We continued to record the male and female together during the breeding season, and the female had a litter of 4 kittens. For the female Iberian lynx (Case #2), we observed interactions with at least three different males during routine field operations in 2022 and 2023, including interactions during mating season.

## 4 DISCUSSION

To the authors' knowledge, these are the first reported cases of spatial use in felids following surgical amputation of an injured limb or a traumatic amputation. Our results provide an insight into the movement and behaviors of amputee felids and are valuable for the recovery of threatened felids and the persistence of currently stable species. Contrary to our hypothesis, none of the individuals differed in the amount of space used or daily distance traveled compared to total ambulatory conspecifics. Only the male Iberian lynx showed a significant decrease in the daily distance traveled, and a smaller home range post‐amputation, but these estimates were still comparable to the movements of conspecific males reported in the literature.

### 4.1 Iberian lynxes

Home‐range estimates for the male Iberian lynx, although in accordance with those found in the literature, showed differences between pre‐ and post‐amputation stages. The main spatial differences between pre‐ and post‐amputation periods for the male Iberian lynx are shown in both home‐range estimates and daily distances, suggesting decreased mobility may be due to the missing forelimb. After forelimb amputation, domestic carnivores face many difficulties in returning to normal behavior since they place more than 60% of their weight on their forelimbs (Cole & Millis, 2017). However, weight recorded at necropsy suggested that the individual adapted well to the new environment where released and was able to hunt wild prey once the supplemental feeding station was not in use. This is in accordance with findings from museum specimens where lynxes with amputated limbs were able to hunt successfully (Garcia‐Perea, 2000) and, to a lesser extent, with an experimental release of an Iberian lynx rehabilitated with a fracture of the proximal left femur epiphysis (Rodríguez et al., 1995). The observed differences in pre‐ and post‐injury spatial use may be due to the physical impediment, but we cannot rule out the effects of an unstable initial territory at reintroduction, since it may take several years to stabilize home ranges during the first years of a lynx reintroduction program (Rueda et al., 2021). The difference may also have been due to the lack of long‐term monitoring resulting in fewer relocations that led to lower estimates or due to the effect of the supplemental feeding station used in the first few weeks (López‐Bao et al., 2008). Lastly, encountering the female lynx just 1 week after release could also have played a role in the male's movement, partially restricting and overlapping his space use to this female's territory. Despite the impairment, the male lynx from our study was also able to naturally interact with a conspecific and successfully reproduce, establishing a potential area for reintroduction of the species.

We do not believe the male Iberian lynx's death was a consequence of the physical impairment. Up to 12.5% of lynxes die from drowning in similar anthropogenic structures (Ferreras et al., 1992), and three individuals (two subadult females in 2016 and one subadult male in 2019) died in artesian wells or irrigation ponds in the same reintroduction area (F. Nájera, Direccion General de Medio Natural y Desarrollo Sostenible, unpublished data). None of these lynxes showed signs of physical impairment at the time of death, suggesting that animals get trapped in these structures and are unable to escape. Irrigation structures, such as irrigation canals, have been reported as the cause of death of 134 vertebrate species, 20.9% of them mammals, in West central Spain during a study period of 13 months (García, 2009). In carnivores, such structures may not only be deadly but also may fragment habitats, disrupt seasonal movements, and hinder gene flow (Baechli et al., 2021; Krausman & Bucci, 2010).

The female Iberian lynx showed signs of adaptation to her injury as observed from body condition scores during the 20‐day time lapse recorded by camera traps. Home‐range estimates from this female are larger than those previously described for the species and sex (Rueda et al., 2021), probably because she was still in a dispersal phase during the first months after release. This also emphasizes that the female did not show difficulties in mobility or territory acquisition after the limb amputation. Although field personnel have not yet observed offspring from this female, interactions with individuals from the opposite sex have only been observed during the last two mating seasons meaning that breeding could occur within the lifespan of the individual despite the physical impairment, as has been observed in other felid species in captivity with a similar limb loss (J. Peña, Jaguar (*Panthera onca*) Reintroduction Project in Parque Iberá, Corrientes, Argentina, Rewilding Argentina, unpublished data).

### 4.2 Bobcat

The bobcat did not show signs of poor adaption despite the missing hind limb, exhibiting size and weight normal for an adult male bobcat in Missouri (Hamilton, 1982). The home‐range size estimated for this individual (during the winter) was much smaller than that estimated for the overlapping resident male across an annual home range. This overlapping individual presented a home range even larger than those reported for other males in Missouri, which may reflect the low density of bobcats in this area (Hamilton, 1982). On the other hand, similar home ranges to those observed for the amputee bobcat have been described (Ferguson et al., 2009). Furthermore, if we consider just winter home ranges in male bobcats from this state, we didn't find significant differences (36.7 km^2^ 100% MCP for the amputated male vs. 41.9 km^2^ for other males; Hamilton, 1982). Although both adult bobcats differed in home‐range size, daily distances covered were similar in both individuals. This may suggest that the regular movements required for daily activities are hindered less by a missing hindlimb than a missing forelimb. This agrees with museum bobcat specimens, where severe femoral fractures that healed over time did not impede survival for long periods of time, probably due to physical and behavioral compensation for this type of injury (Argyros & Roth, 2016).

The short‐term monitoring of this case prevented us from knowing if the individual was able to mate or from exploring differences in space use and home range during spring, summer, and autumn, as these seasons could present distinct movement patterns (Hamilton, 1982). The good body condition recorded during the physical examination as well as during the last photo captured (Figure 6), combined with the home‐range estimates and the daily distances covered, all suggest that this individual adapted adequately despite the major physical limitation.

### 4.3 Snow leopard

For both the pre‐and post‐amputation releases, the daily distances traveled by the snow leopard shortly after release and 3‐month post‐release were similar, suggesting that daily movement of this individual was not subject to a period of habituation to the missing limb. The increase in movement post‐amputation shows that the individual did not have difficulties in his ranging behavior. The home ranges calculated from the seven‐month period of post‐amputation collar data fall within the ranges described in the literature for male snow leopards in the region, although there is extensive individual variation in sizes and data (Rosenbaum et al., 2023). Together, the home‐range estimates, daily distances traveled, and body weight of the snow leopard suggest that the individual compensated adequately for the missing limb.

### 4.4 Other considerations

We note that all lesions in all four individuals had presumably anthropogenic origins, highlighting global human‐felid conflict via three different scenarios on three continents. The injuries of both Iberian lynxes originated from vehicle collisions (confirmed for the male and suspected for the female), while we suspect that both the bobcat's and the snow leopard's injuries were due to traps. The probable human origin of these carnivores' traumatic injuries reflects the reality that wild felids face where habitat fragmentation forces road crossings (Barrientos et al., 2021) or where leg hold traps such as snares are widely used (Belecky & Gray, 2020). For example, at the Nam Et‐Phou Louey National Park (Northern Laos), leopards and tigers have become extirpated principally due to an exponential increase in snares (Johnson et al., 2016; Rasphone et al., 2019).

We hypothesize that the bobcat's lesion may have been from a leg hold trap capture since bobcats are considered furbearers in the state of Missouri and trapping is legally permitted (Missouri Department of Conservation, 2021). We suspect that the snow leopard was illegally captured in a leg hold trap intended for marmots (*Marmot* spp.), a species typically hunted for bush meat in the region. Since marmots are also an important prey for snow leopards (Lyngdoh et al., 2014), it would not be unusual for a snow leopard to get caught in a marmot trap in human‐dominated landscapes. Additionally, during the physical examination, we noted a fresh wound which we considered compatible with gunshot. If a missing forelimb did impair hunting abilities, this could facilitate a shift toward easy‐to‐hunt prey such as livestock. The gunshot lesion could well have been inflicted by local villagers due to retaliation for livestock killing (McCarthy, 2000), and livestock killing could represent an indirect outcome after a severe traumatic injury in a carnivore. We could not validate this hypothesis during the study, but we did register an observation of the snow leopard feeding on a 2‐year‐old horse on November 15, 2017. In the case of large carnivores presenting this type of disability, we suggest future studies that include an analysis of the kills made by these individuals to explore if livestock represented a higher proportion of their diets, which would reflect an increase in conflicts with humans.

## 5 CONCLUSIONS

Due to our small sample size, more studies are necessary to develop a better understanding of the spatial ecology, survival, and behavior of amputee free‐ranging felids. Nonetheless, all individuals studied here adapt well to their traumatic injuries, demonstrating movements and space use behaviors typical of their species. All individuals were able to successfully hunt wild prey to maintain body condition and weight within healthy limits. Similarly, reproductive behavior appeared unaffected by amputation, and we observed breeding behaviors and reproduction, suggesting that these individuals contributed to local populations. These normal movement, foraging, and reproductive behaviors underscore the resilience of amputee‐free‐living felids. Given the decline of many carnivore species, these results indicate that amputee individuals are valuable as functional members of wild carnivore populations. Furthermore, our results justify “second chances” in the wild for rehabilitated animals. We thus suggest that amputee felids may remain free‐living as they contribute to local population persistence and appear to maintain good general health and welfare.

## AUTHOR CONTRIBUTIONS

**Fernando Nájera:** Conceptualization (lead); data curation (lead); formal analysis (equal); investigation (lead); methodology (lead); supervision (lead); visualization (lead); writing – original draft (lead); writing – review and editing (lead). **Stella F. Uiterwaal:** Data curation (equal); formal analysis (lead); investigation (equal); methodology (equal); writing – review and editing (equal). **Elena Crespo:** Methodology (equal); resources (equal); writing – review and editing (supporting). **Rebeca Grande‐Gómez:** Methodology (equal); resources (equal); writing – review and editing (equal). **Juan Francisco Sánchez:** Methodology (equal); resources (equal); writing – review and editing (equal). **Manuel Mata‐Huete:** Methodology (equal); resources (equal); writing – review and editing (equal). **Jamie Palmer:** Methodology (equal); resources (equal); writing – review and editing (equal). **Gabone Iturrarte:** Methodology (equal); resources (equal); writing – review and editing (equal). **Jorge Peña:** Methodology (equal); resources (equal); writing – review and editing (equal). **Bayaraa Munkhtsog:** Methodology (equal); resources (equal); writing – review and editing (equal). **Bariushaa Munkhtsog:** Methodology (equal); resources (equal); writing – original draft (supporting); writing – review and editing (equal). **Andrey D. Poyarkov:** Methodology (equal); resources (equal); writing – review and editing (equal). **Jose A. Hernandez‐Blanco:** Methodology (equal); resources (equal); writing – review and editing (equal). **Dmitry Y. Alexandrov:** Methodology (equal); resources (equal); writing – review and editing (equal). **Naranbaatar Galsandorj:** Methodology (equal); resources (equal); writing – review and editing (equal). **Sharon L. Deem:** Funding acquisition (equal); methodology (supporting); project administration (equal); resources (equal); supervision (supporting); writing – original draft (supporting); writing – review and editing (equal).

## CONFLICT OF INTEREST STATEMENT

The authors declare that they do not have competing interests.

## Supporting information


Appendix S1
Appendix S2

## Data Availability

Data from the snow leopard and bobcat are available from a repository: https://zenodo.org/record/8407626. Data from the Iberian lynx, due to its Endangered status, are available from authors upon reasonable request.
