# Supplementary material for: Insights into the spatial ecology of severely injured free‐living felids: Iberian lynx, bobcat, and snow leopard
Source: Ecol Evol. 2024 Feb 16;14(2):e11000. doi: 10.1002/ece3.11000 (PMC10870331; doi:10.1002/ece3.11000)
Supplement: Supplementary file 1 — Appendix S1 Appendix S2 [file ECE3-14-e11000-s001.docx]

Appendix 1.

Study areas

*Case #1: Male Iberian lynx*

This case occurred in the province of Toledo (Central Spain; Fig. 1), within the reintroduction boundaries designed on the Life+Iberlince Project. This site consisted mainly of private hunting estates, with villages in and around those sites. The altitude ranged between 750–780 m above sea level and temperatures ranged between –4 ºC–38ºC. The landscape was a mixture of open oak woodlands (“dehesa”), scrubs, and cultivated lands. Vegetation was dominated by holm oak *Quercus rotundifolia*, and olive *Olea europea* trees, with a shrub layer of Mediterranean maqui scrubland (e.g., *Retama sphaerocarpa*, *Erica* spp., *Cistus* spp. and *Rosmarinus* spp.) and dense scrub (*Pistacia lentiscus, Quercus coccifera and Flueggea tinctoria)* but, also, open pasture areas. The main land uses included estates managed for large (e.g. red deer -*Cervus elaphus*-, wild boar -*Sus scrofa*-) and small (e.g. European wild rabbit - *Oryctolagus cuniculus*-, red-legged partridge -*Alectoris rufa*-) game hunting, extensive farming (cereal crops and vineyards), and livestock farming.

*Case #2: Female Iberian lynx*

This case also occurred within the Life+Iberlince Project, but was encountered in a different province (Badajoz, Extremadura, Southwestern Spain; Fig.1), and within the reintroduction area known as Hornachos-Matachel Valley. The landscape mainly contained Mediterranean woodland of evergreen oak (*Quercus ilex ballota*), scrubland dominated by *Retama sphaerocarpa* and/or *Cistus ladanifer*, and small patches of open pasture and cropland. This region is characterized by its Mediterranean climate, with very hot, dry summers. The altitude ranged between 213-297 m above sea level, and temperatures ranged between –2 ºC–39ºC. This site consisted mainly of private hunting estates for large and small game, with villages around this area.

*Case #3: Bobcat*

The bobcat in this study was captured as part of the Carnivore Conservation Initiative, a local study of mesocarnivore health and spatial ecology in Saint Louis County, Missouri (USA). Trapping operations took place at Tyson Research Center (TRC; Fig. 1). TRC is an 809 ha. Washington University in St. Louis property, located within the Ozark Border Natural Division. The maximum and minimum elevations were 127 and 233 m, and temperatures ranged between –5 ºC–35ºC. Eighty-five percent of TRC was forested where the main canopy trees were: white oak (*Quercus alba*), red oak (*Quercus rubra*), black oak (*Quercus velutina*) and pignut hickory (*Cmya glabra*). The remaining 15 percent was either open grasslands or service features (Sexton et al. 1997).

*Case #4: Snow leopard*

The snow leopard case arose during the field operations (field surveys, camera trapping, and live trapping) carried out within the framework of the study of Snow leopards in Khosoot Valley, Sutai Khairkhan Mountain, Mongolia (Fig. 1). The Suitai Khairkhan Mountain is located in western Mongolia’s Altai Mountain range, in the Khovd and Gobi-Altai provinces, with an altitude of 4,000 m above sea level. Temperatures ranged between –30 to 35ºC. The dominant vegetation types were alpine, high mountain, and mountain steppe, and the most represented plant families included *Asteraceae, Fabaceae, Rosacea, Caryophyllaceae, Poaceae, Ranunculaceae* (Gundegmaa et al., 2022). Native prey items in this region included: Argali sheep (*Ovis ammon*), Siberian ibex (*Capra siberica*), marmot (*Marmota siberica*), and chukar partridge (*Alectoris chukar*), although consumption of domestic livestock has also been documented (Shehzad et al. 2012).

Appendix 2.

**Detailed case descriptions**

*Case #1: Male Iberian lynx*

In July 2015, an adult male Iberian lynx (“Lucero”) was released in as a part of the Iberian Lynx Reintroduction Program in Toledo (Central Spain). The individual had been tagged prior to his release with a GPS-GSM collar (Microsensory, Fernán Núñez, Spain), recording positions every 4 hours, with the last recorded position occurring on 7 January 2018. Since he was a resident male, tracking of the individual was performed by camera-trapping on a regular basis after that date.

On 22 July 2019, the field team of the Life+IBERLINCE project was alerted of a recent vehicle-lynx collision. Despite the intense search for the individual, the field personnel could only find a broken radio-collar on the road. The collar belonged to “Lucero.” Since the individual could have still been alive, we strategically deployed camera-traps (Moultrie W-900, Birmingham, AL) in areas frequently visited by this individual. We observed this lynx one week later, limping on his left forelimb. On 15 August 2019, a local villager informed the field team that “Lucero” had been accidentally trapped in a chicken coop.

We chemically immobilized the lynx via pole syringe with a combination of dexmedetomidine (Dexdomitor® 0.5mg/ml, Ecuphar; 0.02 mg/kg IM), midazolam (Midazolam Normon® 15mg/3 ml, 0.4 mg/kg IM) and ketamine (Imalgene® 1000, Merial 100 mg/ml, 5 mg/kg), An initial health assessment in the field revealed a severe skin abrasion in the dorsal aspect of the left forelimb, and complicated crown fractures of the mandibular and maxillary left canines. The individual weighed 11.3 kg. On site, we topically cleaned skin wounds with chlorhexidine 0.05%, then administered an anesthetic reversal (atipamezole, Antisedan® 5 mg/ml, Zoetis, 0.2 mg/kg IM) to the individual and transported him to El Chaparrillo Wildlife Rehabilitation Centre (Ciudad Real). Due to the poor body condition, we decided to postpone a further evaluation until the individual was stable and gained weight. During this time the lynx was placed in a quarantine area, a non-steroidal anti-inflammatory drug was administered (Metacam®, meloxicam injectable, Boehringer Ingelheim, 0.2 mg/kg initial then 0.1 mg/kg once daily orally for 5 days) and he was fed whole prey (e.g. domestic rabbits). The lynx was still limping and dragging the left forelimb despite the medication initially administered.

On 5 September 2019, the lynx was again evaluated for the left forelimb lesion. During the physical examination under anesthesia with dexmedetomidine (Dexdomitor® 0.5mg/ml, Ecuphar; 0.02 mg/kg IM), midazolam (Midazolam Normon® 15mg/3 ml, 0.4 mg/kg IM) and ketamine (Imalgene® 1000, Merial 100 mg/ml, 4 mg/kg IM) we took dorso-ventral and lateral radiographs, although no abnormalities were found. We monitored vital signs such as heart rate, respiratory rate, peripheral capillary oxygen saturation (SpO2), and rectal temperature. We administered 5 ml/kg/hr Ringer’s lactate IV fluids (Ringers Braun®) and antibiotics (cefovecin, Convenia®, 80 mg/ml, Zoetis, 8 mg/kg SC). He weighed 12 kg. After the administration of the anesthetic reversal and when the individual was still in the recovery crate, we performed a partial neurological examination to evaluate the postural reaction and sensations. This examination revealed lack of proprioception and superficial and deep pain. Our effort to get a CT scan or MRI to rule out neurological diseases was unsuccessful due to lack of funds and logistical difficulties. A tentative diagnosis of caudal brachial plexus avulsion was made based on the medical history, clinical presentation, and neurological examination. Poor prognosis for the affected limb resulted in a decision to perform a surgical amputation. Due to logistical constraints, the surgery could not be performed until 18 October 2019. For the surgical procedure the lynx was transported to a veterinary referral center in Ciudad Real, then anesthetized with dexmedetomidine-midazolam-ketamine and supplemented with isoflurane anesthesia. During the surgery, he was given 10 ml/kg/hr Ringer’s lactate IV fluids. In addition, we monitored vital signs such as heart rate, respiratory rate, peripheral capillary oxygen saturation (SpO2), rectal temperature, and end-tidal carbon dioxide partial pressure (ETCO2) with a dedicated monitor. The body weight was 10 kg, and there was a severe ulcer on the dorsal aspect of the forelimb that was not observed while in quarantine due to the lack of a camera monitoring system at the facility which, along with the secretive behavior of the individual, prevented clear observation of the limb.

We performed surgical amputation of the proximal half of the humerus uneventfully, and the lynx recovered without complications from the anesthesia and surgery. For the rehabilitation process he was first placed in a 40-m2 quarantine enclosure, monitored via video by a camera trap located inside the enclosure and visually from a distance by the rehabilitation personnel. He was fed dead whole prey (e.g., domestic rabbit, quail, one-day-old chicks). After one week, he was also offered live prey (domestic rabbit) at least 3 times per week. The individual showed no difficulties hunting domestic rabbits, although the enclosure was too small for the rabbit to escape from the lynx. Since we could not fully address mobility in the quarantine enclosure, we built a larger facility (1400 m2) at the center to allow wider movements for the individual to permit live prey to have more space to use while being chased by the lynx. We moved the lynx to the larger enclosure in May 2021, and offered him live prey on a weekly basis to aid in the rehabilitation process and hunting skills. The lynx showed hunting skills in accordance with the species, and he was considered ready to be released into the wild.

On 26 October 2021, he underwent a complete physical examination and sample collection for infectious disease screening under anesthesia. We fitted him with a VHF-GPS-GSM collar (Microsensory, Fernán Núñez, Spain) programmed to provide locations every 4 hours. His weight was 11.85 kg and no clinical signs were found during physical exam. Prior to release, he tested negative for canine distemper virus (CDV), feline leukemia virus (FeLV), feline immunodeficiency virus (FIV), feline calicivirus (FCV), feline coronavirus (FCoV), feline parvovirus (FPV) and feline herspesvirus-1 (FHV-1) by PCR. He also received a vaccination booster against FeLV (FeLV PureVAX, Merial, France).

We selected the release site due to favorable conditions in regard to low intraspecific competition, presence of a resident adult female, prey availability, and habitat suitability. Also, Castilla-La Macha wardens (Albacete province) provided *in situ* monitoring of the individual to: 1) decrease human-caused mortality risk (e.g. illegal poaching) or any other human-related disturbance (e.g. villagers’ visits to the release site); 2) perform visual examinations to monitor body condition; and 3) monitor any relevant behavior such as intraspecific encounters (i.e., male-female) or hunting abilities.

To decrease explorative behavior in search of food, during the first weeks we provided domestic rabbits via a supplementary feeding station, which successfully maintained the lynx near the release site. This location also provided enough opportunities to prey on wild rabbits. This technique has proven to be useful to feed free-ranging lynx and to retain them during long periods under conditions of extremely low prey abundance (Lopez-Bao et al. 2008). Supplementary feeding also has been employed to retain other species of carnivores subjected to release in target areas (Phillips and Parker 1988; Logan et al. 1993). We extended the use of the feeding station over the course of eight weeks, regularly decreasing the number of rabbits provided over the last four weeks to avoid dependence on the feeding station. On 6 February 2022, the mortality signal on the lynx collar alarmed the wardens and they found the individual drowned in a human-made irrigation pond.

*Case #2: Female Iberian lynx*

The second Iberian lynx was a yearling wild-born female Iberian lynx. On 22 February 2019, we video-recorded the individual limping within the boundaries of the reintroduction area of the Hornachos-Matachel Valley. The individual’s gait and right hindlimb appearance resembled a severe fracture. A review of the camera-trap footage revealed that the individual first showed this lesion at least 20 days prior. Surprisingly, the lynx’s body condition improved during that time, meaning that the individual had already adapted to having this disability. The field team successfully captured the lynx via cage traps within two days and brought the lynx to the Iberian lynx captive breeding center in Zarza de Granadilla (Caceres) where emergency surgery was scheduled the same day.

We anesthetized and monitored the individual in a similar fashion as the male lynx case described above. She weighed 4.8 kg. Radiological examination exposed an open fracture of the right tibia and fibula, with necrosis of the right tarsus. Physical examination also revealed skin abrasions in several regions, and several frayed claws, all compatible with a vehicle collision. Due to the grave prognosis for functional use in the affected limb, we uneventfully performed surgical amputation distal to the femur and the lynx recovered without complications from the surgery. She received antibiotics (cefovecin, Convenia®, 80 mg/ml, Zoetis, 8 mg/kg SC) and non-steroidal anti-inflammatories (meloxicam, Metacam,® Boehringer Ingelheim, 0.2 mg/kg SC).

We placed the individual in two quarantine areas (50-m^2^ each) under continuous video monitoring. Since the individual survived in the field during the acute stage of the lesion and did not suffer from weight loss based on the camera-trap footage, we provided live prey (domestic rabbits) daily. During the quarantine, the lynx’s hunting skills were considered normal, and she did not show any signs of struggle while hunting. We kept the individual at the facilities for 40 days and, on 5 April 2019 we anesthetized the lynx to undergo a complete physical examination and to attach a VHF radio-collar (Q-4, Andreas Wagener, Köln, Germany) before release back to the capture site in the field. At this time, the lynx’s weight was 6 kg. Once released we monitored the animal via camera traps and using telemetry performed 1-3 days per week. Locations were obtained by triangulation using a 3-element Yagi antenna (RA-23, Telonics, Mesa, AZ) and hand-held radio telemetry receiver (ICOM America, Kirkland, WA) as previously described (see Rueda et al., 2021).

On 28 June 2022, we captured the lynx for a routine health assessment and changed the radio-collar (Q-7, Andreas Wagener, Köln, Germany). The individual presented with a good body condition score, and body weight was 8.7 kg. During routine field operations in 2022/2023, she had been observed interacting with at least three different males, including interactions during mating season. Table 2 summarizes the main events that occurred during the rehabilitation process of this female Iberian lynx.

*Case #3: Bobcat.*

On 9 February 2022, we captured a male bobcat in a commercial cage trap (Tomahawk model 207, Tomahawk Live Trap Co., Tomahawk, Hazelhurst, WI, USA) baited with road-killed white-tailed deer. We anesthetized the bobcat with a mixture of medetomidine-tiletamine-zolazepam (F. Nájera, UC Davis Wildlife Health Center, unpublished data.). Once under anaesthesia, a routine physical examination revealed a missing right limb below the tibiotarsal joint. Upon further examination, the lesion was noted as completely healed with skin overgrowth for an estimated time of injury at least three months prior. No radiographs were available at the time of the capture to evaluate the appearance of bone structures.

Red blood cell count and serum biochemistry did not reveal any abnormalities. All other physical examination findings were within normal limits. Based on morphology and dentition, the bobcat was considered adult. The bobcat weighed 10.5 kg. We fitted the individual with a GPS-logger (W500, Advance Telemetry Systems, Isanti, Minnesota, USA) programmed to record locations every 4 hours. The individual recovered uneventfully from the anaesthesia, and we released the bobcat at the site of capture the same day.

After one month of tracking via VHF and recording locations by downloading data from the GPS-logger, we lost signal of the individual. One week later, the bobcat was photographed by a camera-trap in a private estate 20 km Southeast of the capture site. Photos from this camera trap site revealed that the individual was in good body condition (Fig. 6). Despite our efforts of terrestrial and aerial telemetry, we were unable to locate the individual again. To allow a comparison between two adult males from the same area but with different conformation, we include a second adult male with all four limbs intact, captured one week earlier at the same site, in this study. Capture, anaesthesia and tagging protocols were the same for both bobcats.

*Case #4: Snow leopard*

On 23 October 2017, we captured an adult male snow leopard in the Khoshoot valley with an Aldrich snare. We performed field anaesthesia by administration via dart of a combination of tiletamine-zolazepam (Zoletil®, Virbac, France, 3 mg/kg IM) and medetomidine (Domitor®, Pfizer, USA, 0.012 mg/kg IM). We reversed medetomidine with atipamezole (Antisedan, Pfizer, USA, 0.1 mg/kg IM). Once under anaesthesia, we observed the right forelimb missing below the proximal humerus. The injury appeared to be from a recent traumatic event based on the stage of wound healing. We suspected that the individual was illegally captured in a leg hold trap intended for marmots (*Marmot* spp.), a typical bush meat in the region. We sutured the skin flap to protect the muscle and bone structures.

This individual also presented a separate, fresh wound of 5.6 mm diameter located in the left lateral of the caudal abdomen, compatible with a gunshot. Despite the leopard’s missing limb, the individual had a good body condition score, with a body weight (44 kg) within normal range for the sex and species (Johansson et al. 2013). We fitted the individual with a GPS-Iridium collar (Lotek Iridium LiteTrack 420, Lotek Wireless Inc., Ontario, Canada) programmed to provide 12 locations per day, that functioned until 5 July 2018. We had previously captured and collared (Lotek GPS-Argos collar, Lotek Wireless Inc., Ontario, Canada) this individual for the first time on 12 November 2016, although at that time the collar malfunctioned and provided locations only until 20 March 2017. At the time of this first capture, the weight was 46 kg and no serious injuries were observed.
